# The incidence and prognosis of nasopharyngeal carcinoma patients with family history

**DOI:** 10.18632/oncotarget.21994

**Published:** 2017-10-24

**Authors:** Yansu Wang, Chunying Shen, Xueguan Lu, Chaosu Hu

**Affiliations:** ^1^ Department of Radiation Oncology, Fudan University Shanghai Cancer Center, Shanghai, China; ^2^ Department of Oncology, Shanghai Medical College, Shanghai, China

**Keywords:** nasopharyngeal carcinoma, family history, incidence, prognosis

## Abstract

**Purpose:**

Epidemiological data showed that nasopharyngeal carcinoma (NPC) was a regional malignancy. It suggested that genetic factor may play an important role in tumorigenesis of NPC. The aim was to investigate the incidence and the prognosis of NPC patients with family history.

**Methods:**

The clinical data of patients with NPC treated in Fudan University Shanghai Cancer Center from January 2008 to December 2012 were reviewed, and the patients with family history were selected. The prognosis of patients with family history was follow-up. The 5-year overall survival (OS), local recurrence-free survival (LRFS), and distant metastasis-free survival (DMFS) were analyzed by Kaplan-Meier and log-rank test. Cox proportional hazard model was used for multivariate analysis.

**Results:**

There were 3.64% (135/3706) NPC patients with family history of NPC. Eighty-three percent (112/135) patients had only one family member suffering from NPC previously, and 74.1% (100/135) patients who had family history only in first-degree family members. Excluding five patients lost to follow-up, 130 patients were eventually used to analyze the prognosis. The 5-year OS, LRFS, and DMFS rates of all patients with family history were 84.1%, 83.4%, and 83.8%, respectively. There were no significant differences of OS, LRFS and DMFS between one relative group and at least two relatives group. In addition, the degree of NPC had no association with OS, LRFS and DMFS, respectively.

**Conclusion:**

Our results showed that there was an incidence rate of 3.64% NPC patients with family history. These patients had a satisfied prognosis, and the prognosis of NPC patients with family history in different degree or numbers of relatives had no significant differences.

## INTRODUCTION

Nasopharyngeal carcinoma (NPC) is one of the most common cancers in areas of Southeast Asia and North Africa, especially in Southern China. The incidence of Southeast Asia was 6.4 per 100,000 for males and 2.0 per 100,000 for females, which is the highest in the world. In 2012, an estimates total of 86,700 cases added to NPC and 50,800 to death [1, 2].

The distinct racial and geographic distribution of NPC suggests a multifactorial cause. Current epidemiological and experimental data identify at least three important etiologic factors: viral, environmental, and genetic [1, 3]. *In viral* factor, Epstein-Barr virus (EBV) has been associated with NPC, especially the nonkeratinizing type. EBV infection may influence the early stages of tumorigenesis in NPC [4-7]. The potential environmental etiologic factors include high consumption of salt-preserved fish and other foods, cigarette smoking, alcohol, occupational exposures, air pollution and wood fire, etc [8-14].

Similar studies reported a significant association between neoplastic prognosis and positive family history, which found better prognosis in breast cancer, colon cancer and gastric cancer [15-18]. In addition, the high incidence of NPC among Southern Chinese and population of Southern Chinese descent suggest a component of genetic susceptibility, and genetic factor may play an important role in tumorigenesis of NPC. The epidemiological investigation also showed that there were many families with high incidence of NPC in endemic areas. However, there were few studies to focus on the incidence and the prognosis for the NPC patients with family history, especially in non-endemic areas. In present study, the aim was to investigate the incidence and the prognosis of NPC patients with family history.

## RESULTS

### The incidence of NPC patients with family history

There were 3706 patients with NPC treated in our hospital during five years. A total of 135 patients were found to have family history of NPC, and the incidence rate was 3.64%. The median age of these 135 patients was 50 years old (ranged: 18 to 77 years). The ratio of male to female was 2.97:1. There were 83.0% (112/135) patients who had only one family member suffering from NPC previously. Seventeen percent patients (23/135) had more than one family members suffering from NPC previously. There were 74.1% (100/135) patients with family history in first-degree relatives and 20.0% (27/135) patients with it in second- or third-degree members. Eight patients (5.9%) had family history in both first- and second-/third-family members, respectively.

### The prognosis of NPC patients with family history

Excluding five patients lost to follow-up, 130 patients were eventually used to analyze the prognosis. The main characteristics of these patients were summarized in Table 1. All patients received radiotherapy, and most of patients (93.1%) received intensity modulated radiotherapy (IMRT). Of the 130 patients, 36 (27.7%) patients were treated with radiotherapy alone. The median radiation doses of the planning target volume were 70 Gy (59.4-76 Gy) to the primary tumor, 66 Gy (54-74.8 Gy) to the regional lymph nodes, 60 Gy (54-74 Gy) to the high-risk clinical target volume and 54 Gy (0-64 Gy) to the low-risk clinical target volume. Ninety-four patients (72.3%) received platinum-based chemotherapy, including induction chemotherapy (n=28, 29.8%), concomitant chemoradiotherapy (n=19), induction chemotherapy followed by adjuvant chemotherapy (n=7), induction chemotherapy followed by concomitant chemoradiotherapy (n=34), concomitant chemoradiotherapy followed by adjuvant chemotherapy (n=5) and induction chemotherapy followed by concomitant chemoradiotherapy followed by adjuvant chemotherapy (n=1). In statistics analysis, we put the patients into four subgroups: first-degree group, second-/third-degree group, one family member group and at least two family members group. First-degree group included the patients with first-degree relatives and both first- and second-/third-degree relatives. Second-/third-degree group enrolled the patients with only second-/third-degree relatives. The main characteristics in various subgroups were listed in Table 2.

**Table 1:** The characteristics of NPC patients with family history (n=130)

Characteristics	No. (%)
Gender	
Male	98 (75.4)
Female	32 (24.6)
Age at diagnosis (years)	
Median (Range)	50 (18-77)
Pathology	
nonkeratinizing carcinoma	130 (100)
T stage	
T1	25 (19.2)
T2	49 (37.7)
T3	27 (20.8)
T4	29 (22.3)
N stage	
N0	26 (20.0)
N1	50 (38.5)
N2	45 (34.6)
N3	9 (6.9)
M stage	
M0	127 (97.7)
M1	3 (2.3)
Clinical stage	
I	9 (6.9)
II	36 (27.7)
III	45 (34.6)
IV	40 (30.8)
Radiation technique	
Conventional radiotherapy	9 (6.9)
IMRT	121 (93.1)
Chemotherapy	
No	36 (27.7)
Yes	94 (72.3)
Number of relatives	
1	110 (84.6)
>=2	20 (15.4)
Degree of relatives	
First-degree	95 (73.1)
Second-/third-degree	27 (20.7)
Both first- and second-/third-degree	8 (6.2)

**Table 2:** Summary of clinical indices for the four subgroups about family history

	Number of relatives		Degree of relatives	
	1	>=2	First-degree	Second-/third-degree
T stage				
T1-T2	64	11	62	12
T3-T4	47	9	41	15
N stage	63	13	60	16
N0-N1	47	7	43	11
N2-N3	110	20	103	27
M stage				
M0	107	20	101	26
M1	3	0	2	1
Clinical stage				
I-II	38	7	37	8
III-IV	72	13	66	19
Chemotherapy				
No	32	4	29	7
Yes	78	16	74	20
Radiation technique				
Conventional radiotherapy	7	2	6	3
IMRT	103	18	97	24

With a median follow-up of 71 months (range: 8-97 months), there were 28 deaths and 50 treatment relapse. Distant metastasis (n=28, 44%) was the major failure pattern, followed by local and regional relapse (n=10, 20%), isolated local relapse (n=10, 20%) and isolated regional relapse (n=8, 16%). The 5-year OS, LRFS, and DMFS rates of all patients with family history were 84.1%, 83.4%, and 83.8%, respectively. Of these, one person afflicted with malignant melanoma during the follow-up after one year. However he was not discovered any recurrence and metastasis about secondary neoplasia.

**Figure 1:**
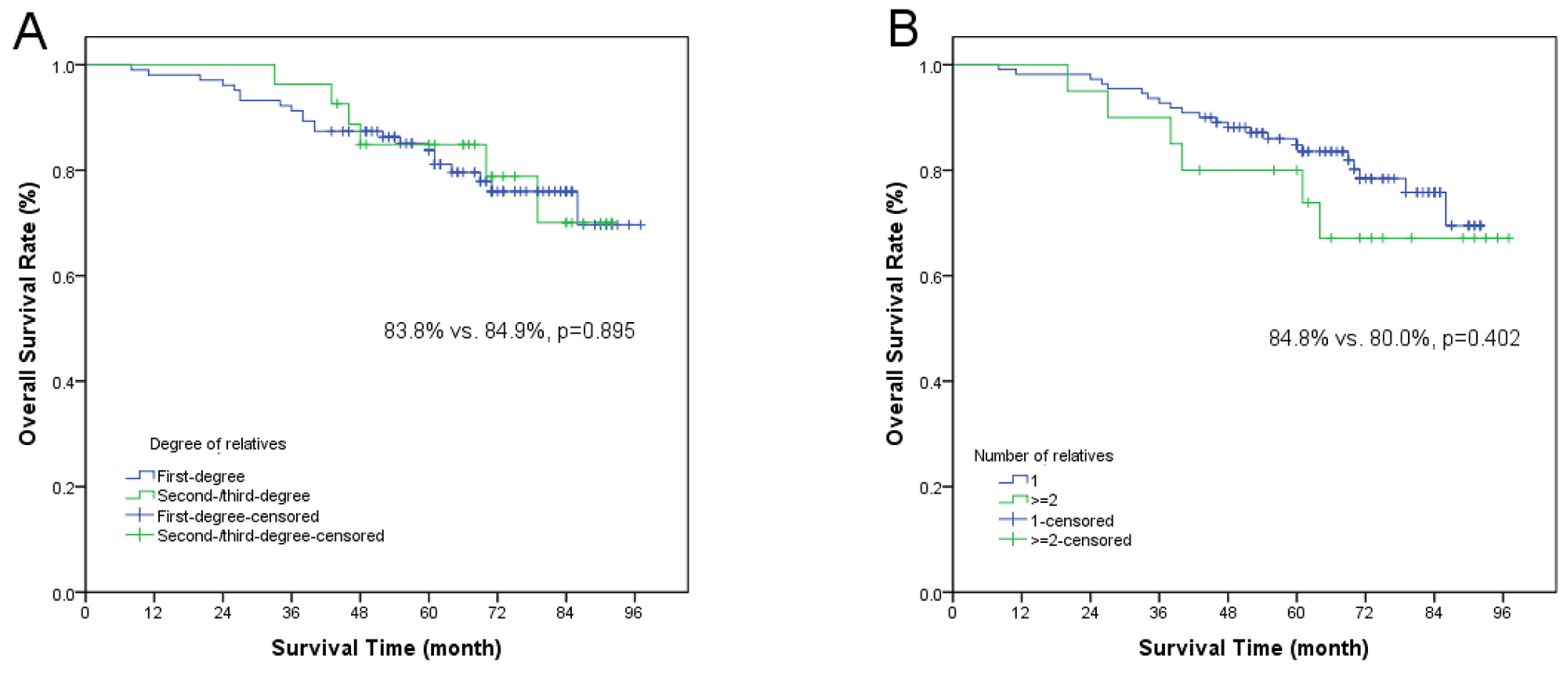

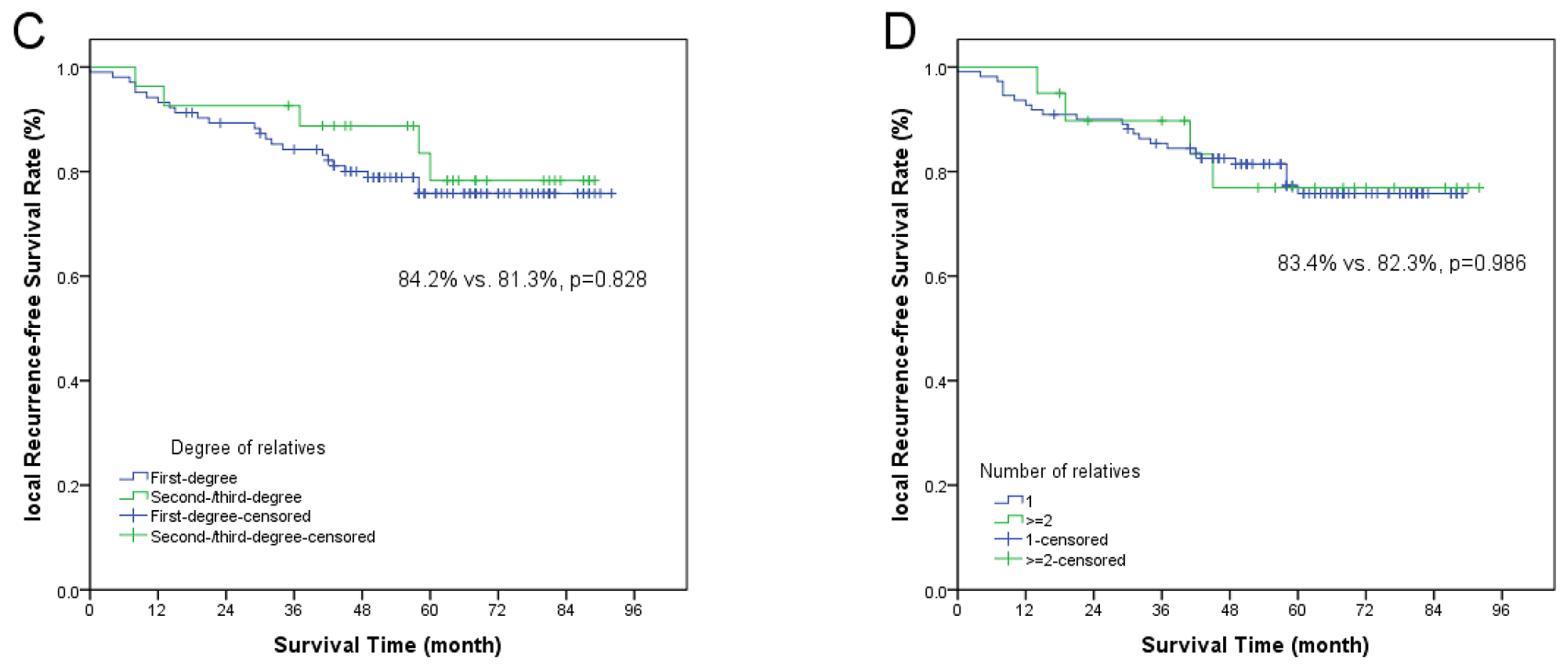

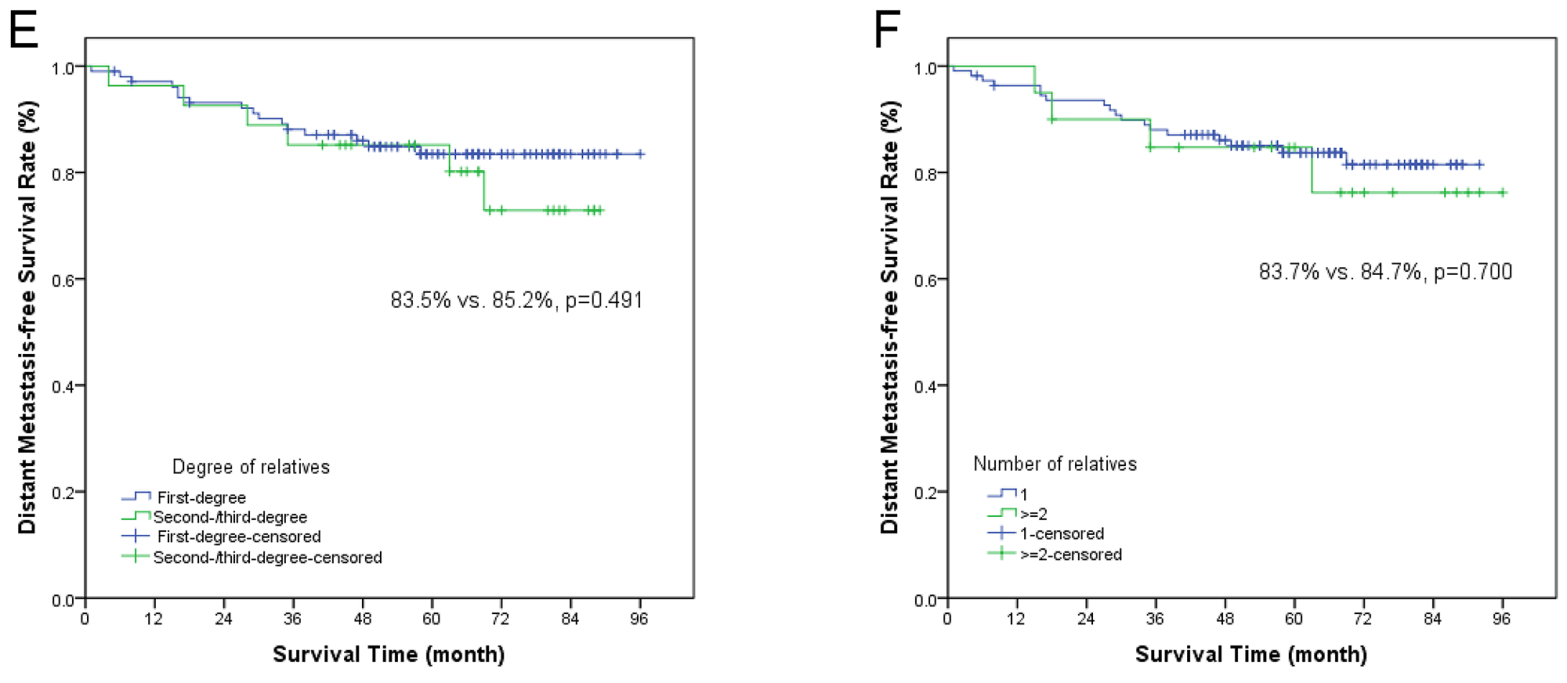
Kaplan-Meier estimate of **(A and B)** overall survival, **(C and D)** local recurrence-free survival and **(E and F)** distant metastasis-free survival in different subgroups.

The univariate analysis results by Kaplan-Meier and log-rank test were listed in Table 3. Stage I-II or T1-T2 was associated with improved OS, LRFS and DMFS. The patients in stage N0-N1 had a reduction of distant metastasis. The locoregional control of IMRT was better than conventional radiotherapy. Male patients and the patients received chemotherapy seemed to have a tendency of distant metastasis. However, there were no significant differences of OS, LRFS and DMFS between one relative group and at least two relatives group. Similarly, the degree of NPC members had no association with OS, LRFS and DMFS, respectively (Figure 1). Multivariate analysis showed that T stage was an independence prognostic factor for OS and LRFS. The patients in early T stage had a decreasing risk of survival and relapse (HR=3.071, 95% CI, 1.054-8.950, *p*=0.04; HR=4.550, 95% CI, 1.038-19.957, *p*=0.045). Compared with conventional radiotherapy, those treated with IMRT had a multivariate HR of 0.270 (95% CI, 0.097-0.749, *p*=0.012) for LRFS (Table 4).

**Table 3:** The 5-year estimated OS, LRFS and DMFS of NPC patients with family history (n=130)

	5-year OS			5-year LRFS			5-year DMFS		
	Rate (%)	χ2	P	Rate (%)	χ2	p	Rate (%)	χ2	p
Age (years)									
<50	89.2	2.979	.084	86.7	1.113	.291	85.4	.324	.569
>=50	78.8			79.5			82.2		
Gender									
Male	81.0	2.127	.145	80.0	1.340	.247	79.6	5.339	***.021***
Female	93.5			93.5			96.9		
Clinical stage									
I-II	97.8	8.263	***.004***	92.7	6.670	***.010***	97.7	7.438	***.006***
III-IV	77.0			78.2			76.5		
T stage									
T1-T2	97.3	14.483	***.000***	94.3	15.127	***.000***	91.2	6.912	***.009***
T3-T4	67.3			68.2			73.9		
N stage									
N0-N1	87.8	1.327	.249	81.8	.000	.987	90.4	5.322	***.021***
N2-N3	78.8			85.6			74.7		
Number of relatives									
1	84.8	.703	.402	83.4	.000	.986	83.7	.148	.700
>=2	80.0			82.3			84.7		
Degree of relatives									
First-degree	83.8	.017	.895	84.2	.047	.828	83.5	.474	.491
Second-/third-degree	84.9			81.3			85.2		
Chemotherapy									
No	91.7	1.899	.168	93.1	3.680	.055	94.1	4.385	***.036***
Yes	81.1			79.7			79.9		
Radiation technique									
Conventional radiotherapy	66.7	3.168	.	40.0	11.907	***.001***	76.2	1.135	.287
IMRT	85.6		075	87.3			84.4		

**Table 4:** Multivariate analysis for clinical factors on prognosis of 130 familial NPC patients

Survival	Factor	*P* value	HR (95%CI)
OS	Clinical stage (I-II/III-IV)	0.327	2.115 (0.473-9.449)
	T stage (T1-T2/T3-T4) *	0.040	3.071 (1.054-8.950)
LRFS	Clinical stage (I-II/III-IV)	0.642	1.591 (0.224-11.302)
	T stage (T1-T2/T3-T4) *	0.045	4.550 (1.038-19.957)
	Radiation technique * (conventional radiotherapy/IMRT)	0.012	0.270 (0.097-0.749)
DMFS	Gender (male/female)	0.146	0.219 (0.028-1.699)
	Clinical stage (I-II/III-IV)	0.747	1.388 (0.190-10.129)
	T stage (T1-T2/T3-T4)	0.160	2.171 (0.737-6.392)
	N stage (N0-N1/N2-N3)	0.199	1.946 (0.704-5.375)
	Chemotherapy (no /yes)	0.523	1.672 (0.346-8.094)

## DISCUSSION

Epidemiological data showed that a few of NPC patients had family history, and it suggested that genetic factor may play an important role in tumorigenesis of NPC. Studies reported that HLA was a significant gene for NPC. Three alleles including HLA-A*1101, HLA-A*0207 and HLA-B*5801 have been confirmed to have a connection with NPC incidence in southeastern China [19-21]. A meta-analysis reported that rs29232 made a contribution to the geographical differences in NPC incidence and suggested that rs29232 might be more associated with NPC in moderate-incidence regions than in high-incidence regions among Han Chinese people. There may be three reasons. First, the heterogeneity of rs29232 was reduced in different regions. Second, rs29232 was independent of HLA-A gene in moderate-incidence regions instead of high-incidence regions. Finally, the odd ratios of rs29232 was higher in moderate-incidence regions than in high-incidence regions [22].

Several studies had reported the incidence of NPC patients with family history in various endemic areas. The incidence rate of familial NPC patients was 9.9%-14.6% in Southern China, including Guangdong and Guangxi province [9, 10, 23, 24]. Another study from Taiwan revealed that 11.0% NPC patients had family history [11]. Furthermore, there were 11.7% NPC patients who had family history with first-degree relatives [24]. In present study, we respectively collected the clinical data of 3706 patients with NPC, and found that 3.64% patients (135/3706) had family history and 2.91% (108/3706) patients had family history with first-degree relatives. The incidence rate of NPC patients with family history in present study was significantly lower compared with the previous studies in endemic areas. The main cause may be that most NPC patients in present study came from Eastern China, a non-endemic area of NPC. These results also suggested that the prevalence of NPC patients with family history was more common in endemic areas than in non-endemic areas.

There were several studies to investigate the prognosis of NPC patients with family history. Cao et al. found that the 5-year OS, LRFS and DMFS rates were 67%, 70% and 76% of sporadic patients, 70%, 83% and 77% of low-frequency familial patients, and 61%, 87% and 64% of high-frequency familial patients. The 5-year LRFS showed significant difference between sporadic and familial NPC patients, whereas the 5-year OS and DMFS revealed no difference [23]. Ouyang et al. suggested that patients with first-degree family history obviously improved OS, LRFS and DMFS. In addition, there was a trend of improving OS, LRFS and DMFS with increasing number of family members [24]. Our study considered that the number of positive relatives and kinship were not associated with OS, LRFS and DMFS, which was contrary to the published researches. One reason is that the patients in the other studies came from endemic areas, while our study was focused on the incidence and the prognosis for familial NPC patients in non-endemic areas. Another reason may be the limit to the small sample.

A limitation of our study is that we didn’t make a case control study. However we compared with the prognosis of 869 NPC patients with IMRT during the same period in our hospital [25]. The 5-year OS, LRFS, and DMFS rates were 84.0%, 89.7%, and 85.6%, which were similar to our study.

Another limitation is the skewed distribution in the subgroups of radiation technique and chemotherapy. The result suggested that the difference between IMRT and conventional radiotherapy was statistically significant. In spite of the result that chemotherapy was associated with DMFS in univariate analysis, it was not statistically significant in multivariate analysis. The main reason that patients received chemotherapy had a lower trend of DMFS than those without chemotherapy might be that most patients with chemotherapy belonged in advanced stage. Another limitation is the skewed distribution in the subgroups of radiation technique and chemotherapy, due to a small number of patients. In spite of the result that chemotherapy was associated with DMFS in univariate analysis, it was not statistically significant in multivariate analysis. The main reason is that most patients with chemotherapy belonged in advanced stage.

## CONCLUSION

Our results showed that there was an incidence rate of 3.64% NPC patients with family history. These patients had a satisfied prognosis after IMRT with or without chemotherapy. The prognosis of NPC patients with family history in different degree or numbers of relatives had no significant differences. However, the major limitation of the present study is that we did not compare the prognosis between patients with and without family history.

## MATERIALS AND METHODS

### Screening of NPC patients with family history

The clinical data of patients with NPC treated in Fudan University Shanghai Cancer Center from January 2008 to December 2012 were reviewed, and the patients with family history were selected. Inclusion criteria were previously untreated, pathologically confirmed NPC, including distant metastasis or not, and receiving whole course of radiotherapy as planed. All investigational sites had Institutional Review Board approval (IRB), and all patients provided voluntary informed consent to participate in the study. The collected data of patients with family history were recorded, including age, gender, pathologic diagnose, clinical stage, therapeutic modalities. All patients were re-staged according to the seventh edition of the UICC/AJCC staging system [26]. The pathology of all the patients enrolled was nonkeratinizing carcinoma (WHO type II, ICD-O 8072/3 [27]). The family members diagnosed with NPC previously were divided into three degrees: first-degree family members (parent, sibling and offspring), second-degree family members (aunt, uncle, nephew, niece and grandparent) and third-degree family members (cousin).

### Follow-up of NPC patients with family history

The follow-up results of these patients were assessed by clinical physical examinations and imaging methods, mainly including nasopharynx and neck MRI or CT scanning, chest CT scanning, abdominal ultrasound and bone scanning. Neoplasm recurrence is defined as local relapse more than six months after first course radiotherapy or re-radiotherapy. Persistent disease is defined as uncontrolled leisions or relapse after first course radiotherapy within six months. The observing end-points were 5-year OS, LRFS and DMFS. The survival duration was defined from the day of treatment to the day of death or last follow-up.

### Statistical analysis

Statistical analysis was performed by using SPSS 19.0. The different survival rates were analyzed by the Kaplan-Meier method and log-rank test. Cox proportional hazard model was used for multivariate analysis by the estimate of hazard ratio (HR) and 95% confidence interval (CI). For all tests, a two-sided p< 0.05 was considered to be significant.

## References

[1] Chang ET, Adami HO (2006). The enigmatic epidemiology of nasopharyngeal carcinoma. Cancer Epidemiol Biomarkers Prev.

[2] Torre LA, Bray F, Siegel RL, Ferlay J, Lortet-Tieulent J, Jemal A (2015). Global cancer statistics, 2012. CA Cancer J Clin.

[3] Jia WH, Collins A, Zeng YX, Feng BJ, Yu XJ, Huang LX, Feng QS, Huang P, Yao MH, Shugart YY (2005). Complex segregation analysis of nasopharyngeal carcinoma in Guangdong, China: evidence for a multifactorial mode of inheritance (complex segregation analysis of NPC in China). Eur J Hum Genet.

[4] Peng H, Chen L, Zhang Y, Guo R, Li WF, Mao YP, Tan LL, Sun Y, Zhang F, Liu LZ, Tian L, Lin AH, Ma J (2016). Survival analysis of patients with advanced-stage nasopharyngeal carcinoma according to the Epstein-Barr virus status. Oncotarget.

[5] Yu KJ, Hsu WL, Pfeiffer RM, Chiang CJ, Wang CP, Lou PJ, Cheng YJ, Gravitt P, Diehl SR, Goldstein AM, Chen CJ, Hildesheim A (2011). Prognostic utility of anti-EBV antibody testing for defining NPC risk among individuals from high-risk NPC families. Clin Cancer Res.

[6] Abdulamir AS, Hafidh RR, Abdulmuhaimen N, Abubakar F, Abbas KA (2008). The distinctive profile of risk factors of nasopharyngeal carcinoma in comparison with other head and neck cancer types. BMC Public Health.

[7] Tay JK, Chan SH, Lim CM, Siow CH, Goh HL, Loh KS (2016). The role of Epstein-Barr virus DNA load and serology as screening tools for nasopharyngeal carcinoma. Otolaryngol Head Neck Surg.

[8] Friborg JT, Yuan JM, Wang R, Koh WP, Lee HP, Yu MC (2007). A prospective study of tobacco and alcohol use as risk factors for pharyngeal carcinomas in Singapore Chinese. Cancer.

[9] Guo X, Johnson RC, Deng H, Liao J, Guan L, Nelson G, Tang M, Zheng Y, de The G, O’Brien SJ, Winkler CA, Zeng Y (2009). Evaluation of non-viral risk factors for nasopharyngeal carcinoma in a high-risk population of Southern China. Int J Cancer.

[10] Ji X, Zhang W, Xie C, Wang B, Zhang G, Zhou F (2011). Nasopharyngeal carcinoma risk by histologic type in central China: impact of smoking, alcohol and family history. Int J Cancer.

[11] Hsu WL, Yu KJ, Chien YC, Chiang CJ, Cheng YJ, Chen JY, Liu MY, Chou SP, You SL, Hsu MM, Lou PJ, Wang CP, Hong JH (2011). Familial tendency and risk of nasopharyngeal carcinoma in taiwan: effects of covariates on risk. Am J Epidemiol.

[12] Nesic V, Sipetic S, Vlajinac H, Stosic-Divjak S, Jesic S (2010). Risk factors for the occurrence of undifferentiated carcinoma of nasopharyngeal type: a case-control study. Srp Arh Celok Lek.

[13] Xie SH, Yu IT, Tse LA, Au JS, Lau JS (2015). Tobacco smoking, family history, and the risk of nasopharyngeal carcinoma: a case-referent study in Hong Kong Chinese. Cancer Causes Control.

[14] Ren ZF, Liu WS, Qin HD, Xu YF, Yu DD, Feng QS, Chen LZ, Shu XO, Zeng YX, Jia WH (2010). Effect of family history of cancers and environmental factors on risk of nasopharyngeal carcinoma in Guangdong, China. Cancer Epidemiol.

[15] Mohammed SN, Smith P, Hodgson SV, Fentiman IS, Miles DW, Barnes DM, Millis RR, Rubens RD (1998). Family history and survival in premenopausal breast cancer. Br J Cancer.

[16] Malone KE, Daling JR, Doody DR, O’Brien C, Resler A, Ostrander EA, Porter PL (2011). Family history of breast cancer in relation to tumor characteristics and mortality in a population-based study of young women with invasive breast cancer. Cancer Epidemiol Biomarkers Prev.

[17] Chan JA, Meyerhardt JA, Niedzwiecki D, Hollis D, Saltz LB, Mayer RJ, Thomas J, Scafer P, Whittom R, Hantel A, Goldberg RM, Warren RS, Bertagnolli M, Fuchs CS (2008). Association of family history with cancer recurrence and survival among patients with stage III colon cancer. JAMA.

[18] Han MA, Oh MG, Choi IJ, Park SR, Ryu KW, Nam BH, Cho SJ, Kim CG, Lee JH, Kim YW (2012). Association of family history with cancer recurrence and survival in patients with gastric cancer. J Clin Oncol.

[19] Lancaster AK, Single RM, Solberg OD, Nelson MP, Thomson G (2007). PyPop update–a software pipeline for large-scale multilocus population genomics. Tissue Antigens.

[20] Cao SM, Simons MJ, Qian CN (2011). The prevalence and prevention of nasopharyngeal carcinoma in China. Chin J Cancer.

[21] Solberg OD, Mack SJ, Lancaster AK, Single RM, Tsai Y, Sanchez-Mazas A, Thomson G (2008). Balancing selection and heterogeneity across the classical human leukocyte antigen loci: a meta-analytic review of 497 population studies. Hum Immunol.

[22] Su WH, Chiu CC, Yao Shugart Y (2015). Heterogeneity revealed through meta-analysis might link geographical differences with nasopharyngeal carcinoma incidence in Han Chinese populations. BMC Cancer.

[23] Cao SM, Chen SH, Qian CN, Liu Q, Xia YF (2014). Familial nasopharyngeal carcinomas possess distinguished clinical characteristics in southern China. Chin J Cancer Res.

[24] Ouyang PY, Su Z, Mao YP, Liang XX, Liu Q, Xie FY (2013). Prognostic impact of family history in southern Chinese patients with undifferentiated nasopharyngeal carcinoma. Br Janc Cancer.

[25] Ou X, Zhou X, Shi Q, Xing X, Yang Y, Xu T, Shen C, Wang X, He X, Kong L, Ying H, Hu C (2015). Treatment outcomes and late toxicities of 869 patients with nasopharyngeal carcinoma treated with definitive intensity modulated radiation therapy: new insight into the value of total dose of cisplatin and radiation boost. Oncotarget.

[26] Edge SB, Byrd DR, Compton CC, Fritz AG, Greene FL, Trotti A (2010). AJCC Cancer Staging Handbook from the AJCC Cancer Staging Manual.

[27] Thompson L (2006). World Health Organization classification of tumors: pathology and genetics of head and neck tumors. Ear Nose Throat J.

